# Identifying Optimal Prostate Biopsy Strategy for the Detection Rate of Clinically Significant Prostate Cancer: A Systematic Review and Meta-Analysis of Randomised Controlled Trials (RCTs) in Biopsy-Naïve Population

**DOI:** 10.3390/cancers17030458

**Published:** 2025-01-29

**Authors:** Wafa D. Aloufi, Abdulrahman Al Mopti, Anas Al-Tawil, Zhihong Huang, Ghulam Nabi

**Affiliations:** 1 Division of Imaging Sciences and Technology, School of Medicine, Ninewells Hospital, University of Dundee, Dundee DD1 9SY, UK; analmopti@nu.edu.sa (A.A.M.); g.nabi@dundee.ac.uk (G.N.); 2 Department of Radiological Sciences, College of Applied Medical Sciences, Taif University, Taif 21944, Saudi Arabia; 3 Radiology Department, College of Applied Medical Sciences, Najran University, Najran 55461, Saudi Arabia; 4 Digital Intelligence, Al Olaya District, Riyadh 12382, Saudi Arabia; anas@digitalintelligence.sa; 5 School of Science and Engineering, University of Dundee, Dundee DD1 4HN, UK; z.y.huang@dundee.ac.uk

**Keywords:** prostate cancer, image guided biopsy, ultrasound, MRI, prostate cancer sampling, random systematic biopsy, meta-analysis

## Abstract

Background: The growing role of multiparametric MRI (mpMRI) and MRI-targeted biopsy (MRI-TBx) suggests they may replace random systematic biopsy (SBx), specifically detection and subsequent treatment of clinically significant prostate cancer (csPCa). Objectives: To perform a systematic review and meta-analysis of randomised controlled trials (RCTs) comparing the detection rates (DR) of csPCa using MRI-TBx alone, SBx alone, or their combination in biopsy naïve patients suspected of having prostate cancer (PCa). Methods: PubMed, MEDLINE, Embase, and the Cochrane Library were searched up to 23 March 2023, for RCTs comparing PCa DR between biopsy strategies in patients with suspected prostate cancer. Detection rates were pooled using random/fixed effect models, and the study quality was assessed using the Cochrane risk of bias revised tool. Results: Ten RCTs (involving 3646 patients) were analysed, revealing that the combined biopsy method achieved higher overall csPCa DR compared to the SBx method alone (RR = 1.40 [95% CI = 1.15–1.71] and 1.47 [95% CI = 1.13–1.92], respectively). However, there was no significant difference in DR for clinically insignificant prostate cancer (ciPCa) between the two methods. Conclusions: This review concludes that MRI-TBx and SBx detect overall and clinically significant prostate cancer (csPCa) better than SBx alone. The variety of factors requires cautious interpretation, yet these findings are the strongest evidence. The combination technique is recommended for biopsy-naïve groups, but more study is needed to optimise execution and overcome uncertainties.

## Simple Summary

Prostate cancer is a common concern for men, and accurate diagnosis of clinically significant cases is essential for effective treatment. Traditional random biopsies may miss significant cancers or lead to overdiagnosis of insignificant ones. This review analysed data from ten high-quality clinical trials and found that combining MRI-targeted biopsy with systematic biopsy improves the detection of overall and clinically significant prostate cancer compared to systematic biopsy alone. While the findings represent the strongest evidence to date, careful interpretation is required due to varying factors across studies. The combined approach is recommended for biopsy-naïve patients, but further research is needed to refine its use and address remaining uncertainties.

## 1. Introduction

Prostate cancer was reported as the second most common cancer in men in 2020 [1] and is predicted to affect 299,010 new cases and kill 35,250 men by 2024 [2]. The traditional diagnostic pathway to determine the presence or absence of PCa includes the Prostate-Specific Antigen (PSA) blood test, digital rectal exam (DRE), and SBx. Typically, SBx involves obtaining 10–12 cores guided by transrectal ultrasonography (TRUS) [3]. However, the TRUS biopsy has limitations, such as limited detection of csPCa and the potential for overdiagnosis of ciPCa [4], along with the risks of side effects due to the high number of biopsy cores sampled [5].

Multi-parametric magnetic resonance imaging has gained prominence as a diagnostic method in patients suspected of having PCa [6]. Prostate biopsy cores can be guided to more precise regions in the prostate using MRI data. There are three primary image-based strategies for MRI-TBx [7]: (1) visual estimation or cognitive targeting (MRI-CB) in which the region of interest (ROI) is determined before biopsy and the biopsy operator estimates where it might be on an ultrasound image [7]; (2) software-assisted fusion (MRI-FB) to identify and draw the ROI on MR images before a biopsy, and then superimposing these ROIs on ultrasound images of the prostate during the biopsy [7]; and (3) in-bore MRI target biopsy (MRI-IB), which requires performing the biopsy within the MRI scanner, guided by MR imaging obtained immediately after each needle placement [7].

Given the prominence of mpMRI and MRI-TBx as emerging diagnostic approaches and the recognised limitations of the traditional SBx method, an updated systematic analysis and comparison of these techniques is essential. Recent clinical report results comparing the two methods, prominent examples of such trials include PRECISION [8], PROMIS [4], and MULTIPROS [9]. Some clinical trials compared SBx and MRI-TBx with SBx for PCa detection showing that combined MRI-TBx and SBx had higher DR compared to the SBx for csPCa [10,11,12,13,14]. However, other clinical trials showed that MRI-TBx could detect more csPCa compared to SBx [9,15,16]. The most recently published systematic review by Xie et al., 2022 [17], observed that MRI-TBx detects more csPCa and high-risk PCa patients and fewer ciPCa ones than SBx. MRI-TBx combined with SBx improve PCa detection but doesn’t reduce ciPCa detection. Since then, more reports have been published involving biopsy-naïve populations. In view of this, further investigation and review of recent evidence is necessary to ascertain the best approach or approaches for detecting csPCa and ciPCa. Therefore, we performed a systematic review and meta-analysis of recently published RCTs to compare the DR of csPCa, ciPCa, and overall DR between:

- the MRI-TBx and the SBx;
- the combined strategy (MRI-TBx + SBx) to the SBx alone;
- the combined strategy (MRI-TBx + SBx) to the MRI-TBx alone.

## 2. Materials and Methods

The study adhered to PRISMA guidelines [18] and was registered in the PROSPERO database (CRD42023421067). Our aim was to systematically review RCTs comparing DR of PCa using MRI-TBx alone, SBx alone, or a combination in biopsy-naïve patients.

### 2.1. Literature Search

PubMed, MEDLINE, Cochrane Library, Embase, clinicaltrials.com, and Google Scholar, along with the reference lists of included studies, were searched for relevant studies using various synonyms, keywords, and MESH terms with no time or language restrictions. The key terms included “prostate biopsy” AND “MRI “ AND “ultrasound” (Supplementary Materials).

### 2.2. Inclusion and Exclusion Criteria

The inclusion criteria were:

- randomised controlled trials for patients with PCa suspicion based on an elevated PSA level and/or abnormal DRE and/or positive MRI scan.
- patients without a prior biopsy (naïve) and studies comparing SBx with MRI-TBx and reporting DR of overall PCa, csPCa, and ciPCa were included.

### 2.3. The Exclusion Criteria Were

- studies were excluded if the participants were non-biopsy-naïve patients.
- review articles, retrospective studies, abstracts, meeting reports, conference papers, ongoing trials, and case reports were also excluded.

The study selection was performed by one reviewer (W.D.A.) and confirmed by two additional reviewers (G.N., A.A.M.).

### 2.4. Data Extraction and Quality Assessment

The articles were imported into ENDNOTE X9, and after title and abstract screening, the full text of relevant papers was reviewed using Covidence review manager tool. The MACRO Excel sheet tool was used for the Cochrane risk of bias revised tool (ROB-2). The following data were collected: (1) authors, year, enrolment period, institution, and country; (2) number of patients, age, serum PSA level, prostate volume, and csPCa definition; (3) MRI magnet strength, sequences, coil type, PI-RADS threshold, and type of MRI; and (4) number of biopsy cores, route, Gleason scoring, and DR.

### 2.5. Data Synthesis and Analysis

A meta-analysis was performed to evaluate the DR of csPCa, ciPCa, and overall PCa across three comparisons, as stated above. A *p*-value < 0.05 was considered statistically significant. Heterogeneity within studies was assessed, with values categorised as insignificant (0–40%), moderate (30–60%), substantial (50–90%), and considerable (75–100%). Forest plots were utilised to visualise the aggregated estimates and 95% confidence intervals. Review manager was employed with a random/fixed effect model to pool DR and relative detection rates. Subgroup analyses were used to decrease the heterogeneity.

## 3. Results

### 3.1. Study Selection

**Figure 1:**
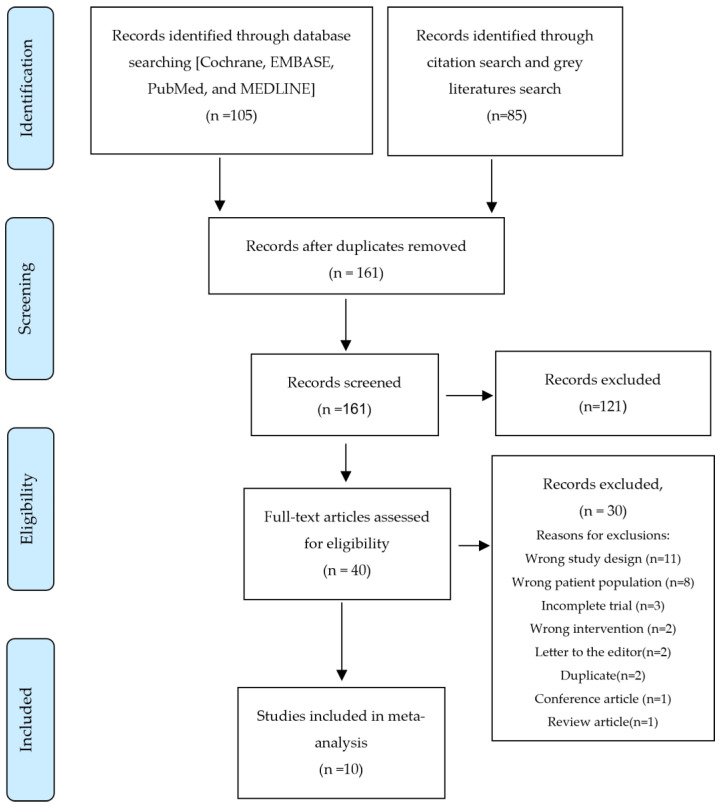
PRISMA Flow Diagram.

The systematic search initially retrieved 190 articles. Following the elimination of duplicates, title and abstract screening, and full-text review, ten RCTs were considered suitable for meta-analysis and systematic review (Figure 1).

### 3.2. Characteristics of the Included Studies

Table 1 and Table 2 display the characteristics of the included studies, as well as MRI and biopsy characteristics, all of which were RCTs conducted between 2011 and 2023. The sample sizes ranged from 85 to 1140 biopsy-naïve patients aged 40 to 82 years old, with only two trials [19,20] conducted at multi-centre locations. One trial [10] did not report the MRI scanner used for mpMRI examination, while all other trials used either 1.5-T or 3-T scanners. Seven studies [9,10,11,12,15,19,20] utilised MRI-FB, while three used MRI-CB [13,14,16]. No study used the MRI-IB method. All trials employed mpMRI, except for [15], used bi-parametric MRI (BpMRI). The most frequent lesion categorisation system (*n* = 7) [9,10,11,12,15,19,20] was the Prostate Imaging Reporting and Data System (PI-RADS) with a score of ≥ 3 as the standard. MRI-TBx typically involved 1–6 cores per lesion, whereas the SBx consisted of 10–12 cores. Three studies used an endorectal coil during prostate imaging [11,13,19], while five did not [9,14,15,16,20]. More detailed patient information can be found in Supplementary Materials Table S1 [9,10,11,12,13,14,15,16,19,20].

### 3.3. Quality of Evidence and Publication Bias

**Figure 2:**
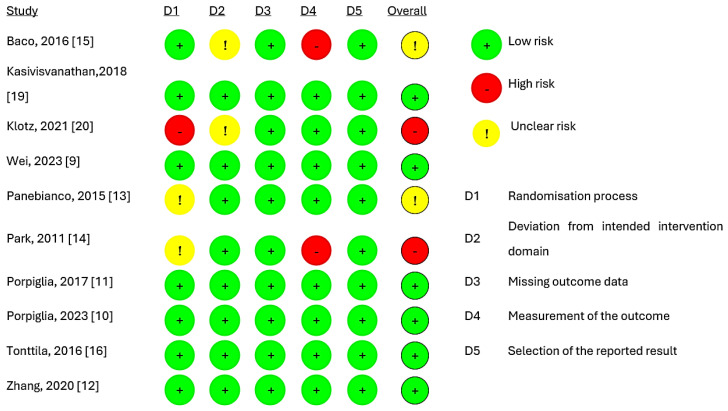
Risk of bias assessment for studies [9,10,11,12,13,14,15,16,19,20].

Figure 2 presents the quality of evidence from the included studies, indicating that six studies [9,10,11,12,16,19] had a low risk of bias. However, two trials [13,15] raised concerns, while two trials [14,20] exhibited a high risk of overall bias. The main reason for this was the absence of details regarding randomisation and the blinding of the pathologic evaluation of biopsy tissues. The assessment of publication bias was not completed due to the inclusion of only ten studies.

### 3.4. Meta-Analysis Results:

#### 3.4.1. Combined Strategy (MRI-TBx + SBx) Versus SBx

**Figure 3:**
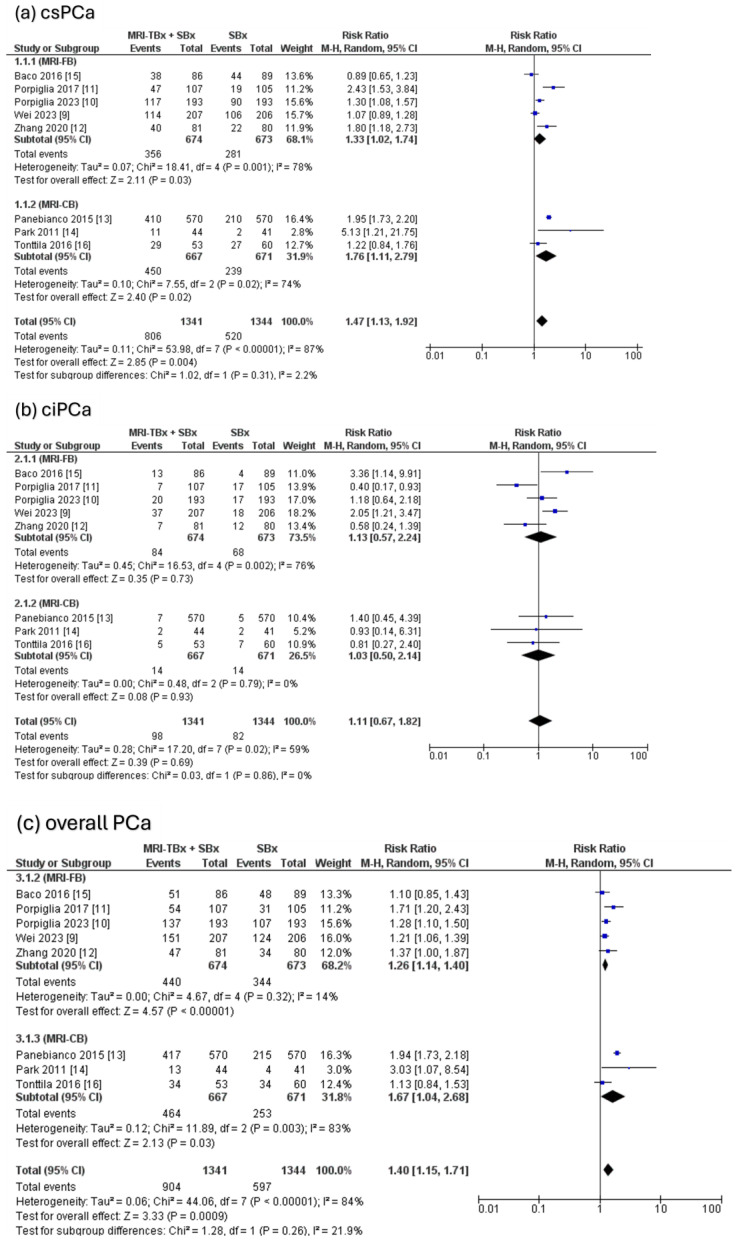
Combined strategy (MRI-TBx + SBx) versus SBx [9,10,11,12,13,14,15,16].

The combined strategy showed a significantly higher DR of csPCa compared to the SBx method alone (RR = 1.47, 95% CI = 1.13, 1.92, *p* = 0.004, I^2^ = 87%, Figure 3a) but there were no significant differences in the DR of ciPCa using the combined strategy compared to the SBx alone (RR = 1.11, 95% CI = 0.67, 1.82, *p* = 0.69, I^2^ = 59%, Figure 3b). For the overall PCa, the combined strategy significantly increased the DR of overall PCa compared to SBx alone (RR = 1.40, 95% CI = 1.15, 1.71, *p* < 0.0009, I^2^ = 84%, Figure 3c).

#### 3.4.2. MRI-TBx Versus SBx

**Figure 4:**
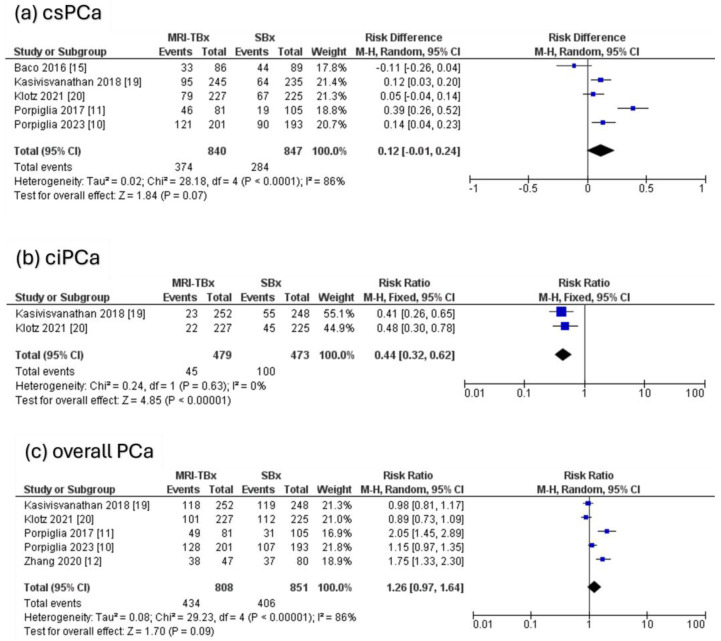
MRI-TBx versus SBx [10,11,12,15,19,20].

Five RCTs were analysed, revealing that there were no significant differences in the DR of csPCa using MRI-TBx compared to SBx (RR = 0.12, 95% CI = 0.01,0.24, *p* = 0.07, I^2^ = 86%, Figure 4a). Two RCTs were analysed for ciPCa, showing that in contrast to MRI-TBx (CB or FB), SBx demonstrated a considerably higher DR of ciPCa (RR = 0.44, 95% CI = 0.32, 0.62, *p* < 0.00001, I^2^ = 0%, Figure 4b). Five RCTs were analysed for the overall PCa and revealed no significant differences in the DR of PCa between methods (RR = 1.26, 95% CI = 0.97, 1.64, *p* = 0.09, I^2^ = 86%, Figure 4c).

#### 3.4.3. Combined Strategy (MRI-TBx + SBx) Versus MRI-TBx

**Figure 5:**
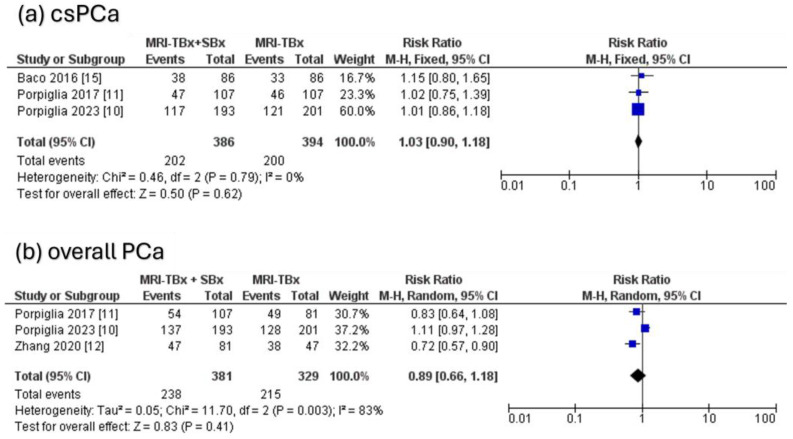
Combined strategy (MRI-TBx + SBx) versus MRI-TBx [10,11,12,15].

For csPCa, the three RCTs analysed indicated no significant difference in the DR between the combined strategy (MRI-TBx + SBx) and the MRI-TBx method (RR = 1.03, 95% CI = 0.90, 1.18, *p* = 0.62, I^2^ = 0%, Figure 5a). For ciPCa, the DR using MRI-TBx were not reported in all trials, preventing the calculations. There was no significant difference in overall PCa DR between MRI-TBx + SBx and MRI-TBx method alone (RR = 0.89, 95% CI = 0.66, 1.18, *p* = 0.41, I^2^ = 83%, Figure 5b).

### 3.5. Multiple Subgroup Analyses for csPCa

Table 3 illustrates the relative DR of csPCa across various subgroup analyses showing that the combined strategy (MRI-TBx + SBx) detected significantly more csPCa than SBx alone when utilising a 3T MRI machine (RR = 1.59 [1.16, 2.16], *p* = 0.004, I^2^ = 77%) and endorectal coil (RR = 1.99 [1.77, 2.24], *p* = 0.00001, I^2^ = 0%). Additionally, significantly improved DR were observed with ≥3 sample cores in the MRI-TBx compared to ≤2 cores/lesion (RR = 1.42 [1.14, 1.77], *p* = 0.002, I^2^ = 61%). Due to limited data and a small number of studies, this was the only feasible subgroup analysis.

## 4. Discussion

The traditional diagnostic strategy for PCa relied on SBx until the recent adoption of MRI for guided targeted biopsies. This systematic review exclusively based on RCTs suggests that the combined strategy of MRI-TBx and SBx may outperform SBx alone, potentially leading to increased diagnoses of csPCa and overall PCa. However, when comparing MRI-TBx to SBx, there was no significant difference in detecting overall and csPCa between the two methods. Though, SBx detected more ciPCa than MRI-TBx, resulting in overdiagnosis of ciPCa. Furthermore, when comparing the combined strategy (MRI-TBx + SBx) with MRI-TBx alone, no significant difference was observed in the DR overall and csPCa. Nevertheless, there was not enough data to analyse the DR for ciPCa, as it was not reported in all studies. Subgroup analyses (Table 2) indicated that the combined strategy detected more csPCa compared to SBx, particularly when using a 3-Tesla MRI machine, an endorectal coil, and a higher number of sample cores per lesion (≥3/lesion).

The study findings agreed with Xie et al. 2022 [17]. A systematic review of the combined approach (MRI-TBx + SBx) over SBx alone shows increased overall and csPCa detection rate. The detection rate of csPCa with the MRI-TBx approach alone was not significantly different from theirs. Our focus on biopsy-naïve individuals may explain the differences in the findings from Xie et al. [17], who included patients with prior-negative biopsy. The systematic review by Hu et al., 2020 [21], focusing on RCTs with a mixed sample (prior negative biopsy and biopsy naïve), found no significant difference between MRI-TBx and SBx. Our results match theirs except for ciPCa, where SBx detects more cases than MRI-TBx. Additionally, Hu et al., 2020 [21] combined trials with varied designs, including Kasivisvanathan et al., 2018 [19], which used MRI-TBx in one arm and SBx in the other, which may have caused biases. This differs from most studies that used the combined MRI-TBx + SBx in one arm and SBx alone in the other, reporting DR appropriately. Moreover, the review by Woo et al. 2019 [22] of mixed-population RCTs compared the MRI-stratified pathway (mpMRI scan followed by MRI-TBx) to the SBx pathway, supporting the findings by Hu et al. [21]. However, Woo et al., 2019 [22] included trials with diverse designs and did not address SBx in the MRI-stratified pathway, which may have biased their results.

There are notable limitations and differences between MRI-TBx strategies that can impact the accuracy and consistency of the procedure. The MRI-CB technique, for example, is highly dependent on the skill and experience of the operator, introducing the possibility of human error [23]. In contrast, the MRI-FB technique improves with accumulated experience but still relies heavily on the quality of alignment and is influenced by the learning curve, with expertise and teamwork being essential factors in enhancing its accuracy [24]. The MRI-IB is the most precise method but is more expensive and, as a result, less commonly used [25]. A 2017 review [26] compared the detection rates of csPCa among three MRI-targeted biopsy techniques, demonstrating that MRI-IB exhibits superior overall PCa detection compared to MRI-CB. MRI-FB and MRI-IB showed comparable DR. A 2022 review [27] reported no statistically significant differences among the three MRI-TBx techniques. Moreover, a study [28] comparing PI-RADS 3–5 and PI-RADS 4–5 as thresholds for targeted prostate biopsy reported that limiting biopsies to PI-RADS 4–5 lesions improves the performance of mpMRI, particularly in detecting aggressive prostate cancers. The PI-RADS 3 threshold may lead to a higher percentage of patients undergoing unnecessary biopsy procedures despite not having clinically relevant PCa, highlighting the need for improved MRI-based biopsy techniques. Additionally, recent studies [29,30,31] have focused on using risk calculators, incorporating prostate volume and PSA density (PSAD), to enhance MRI-based screening for csPCa. A systematic review [32] demonstrated that PSAD improves detection, especially in patients with negative or equivocal MRI findings, like PI-RADS 3 lesions. Lower PSAD values were associated with low probabilities of csPCa, suggesting that biopsy may not be necessary. These findings highlight the importance of integrating prostate volume and PSAD into clinical decisions, improving csPCa detection and minimising unnecessary biopsies. At present, there is no consensus regarding the optimal strategy for targeted biopsy, and the subject remains a topic of debate.

This systematic review has several strengths, including its exclusive focus on RCTs to ensure robust methodology and high-quality evidence for evaluating the efficacy of MRI-TBx and SBx in diagnosing PCa. By intentionally including a biopsy-naïve population, it enhances the relevance of findings to real-world clinical scenarios. Additionally, the removal of language and time restrictions allows for a thorough global analysis, incorporating both transrectal and transperineal biopsy methods to account for variability in clinical practices. This review provides novel insights by incorporating recent evidence, comprehensively analysing key biopsy comparisons, addressing variations in techniques, and conducting detailed subgroup evaluations with a standardised approach. Together, these factors contribute to more clinically relevant and reliable findings while building on prior research.

Nonetheless, this review had certain limitations related to participant numbers, statistical power, and inconsistencies in study designs due to variations in biopsy procedures and MRI scan sequences. The diversity in trial designs and protocols across hospitals, coupled with challenges such as inconsistent definitions of csPCa and PI-RADS thresholds contributed to the complexity of result interpretation. Additionally, unmeasurable variables, such as clinical staff expertise, may introduce confounding factors affecting result reliability. None of the eligible trials included MRI-IB, limiting the scope to specific MRI-TBx strategies and potentially impacting the comprehensiveness of the findings. Therefore, future research should consider increasing participant numbers for enhanced statistical power, prioritise robust randomisation and allocation concealment, standardise biopsy procedures, add the MRI-IB to the trial arms, investigate the impact of variables like clinical staff expertise on outcomes, and focus on the long-term precision and accuracy of MRI-TBx and SBx.

The findings of this review suggest that the combined strategy of MRI-TBx and SBx may improve the detection of overall and csPCa compared to the SBx alone. Clinicians may consider incorporating MRI-TBx alongside SBx in biopsy-naïve patients suspected of having PCa to enhance diagnostic accuracy.

## 5. Conclusions

In conclusion, this review demonstrates that the combined approach of MRI-TBx and SBx achieves higher detection rates for overall and csPCa compared to SBx alone. While the complexity of variables necessitates cautious interpretation, these findings represent the strongest available evidence to date. As such, the combined strategy should be considered the preferred approach for biopsy-naïve populations, with further research needed to optimise its implementation and address remaining uncertainties.

## Figures and Tables

**Table 1:** Studies characteristics.

Study	Multi or Single Centre	Sample Size	Investigation Arm	Comparator Arm
			Arm (1)	Arm (2)
Baco 2016 [15]	Single	175	(MRI-TBx) + 12-core (TRUS-Bx)	12-core (TRUS-Bx) + target core on palpable lesions
Kasivisvanathan 2018 [19]	Multi	500	(MRI-TBx)	10–12-core (TRUS-Bx)
Klotz 2021 [20]	Multi	453	(MRI-TBx)	12-core (TRUS-Bx)
Wei 2023 [9]	Single	413	(MRI-TBx) + 12-core (TRUS-Bx)	12-core (TRUS-Bx)
Panebianco 2015 [13]	Single	1140	14-core (TRUS-Bx) + (MRI-TBx)	14-core (TRUS-Bx)
Park 2011 [14]	Single	85	(MRI-TBx) +10–12-core (TRUS-Bx)	10–12-core (TRUS-Bx)
Porpiglia 2023 [10]	Single	394	(MRI-TBx)	(MRI-TBx) + 12-core (TRUS-Bx)
Porpiglia 2017 [11]	Single	212	(MRI-TBx) + 12-core (TRUS-Bx)	12-core (TRUS-Bx)
Tonttila 2016 [16]	Single	113	(MRI-TBx) +10–12-core (TRUS-Bx)	10–12-core (TRUS-Bx)
Zhang 2020 [12]	Single	161	(MRI-TBx) + 12-core (TRUS-Bx)	12-core (TRUS-Bx)

RCT = randomised controlled trial; MRI-TBx = magnetic resonance imaging targeted biopsy; TRUS-Bx = transrectal ultrasound biopsy.

**Table 2:** MRI and biopsy characteristics.

	MRI Properties					Biopsy Properties			
Study	Type of MRI Target Methods	PI-RADS Score	MRI Machine Field Strength (Tesla)	mpMRI Sequences	Endorectal Coil	MRI-TBx Cores Number	(TRUS-Bx) Cores Number	Biopsy Approach	Definition of Clinically Significant PCA
Baco 2016 [15]	MRI-fusion TBx	PI-RADS ≥ 3	1.5-T (Siemens)	T2, ADC	Without	2 per lesion	12 cores	Transrectal	MCCL ≥ 5 mm for GS 6—disease or any MCCL for GS ≥ 7 disease
Kasivisvanathan 2018 [19]	MRI-fusion TBx; MRI cognitive TBx	PI-RADS ≥ 3	1.5-T or 3.0-T/(Philips, GE, Siemens)	T2, DWI, DCE	With or without	4 per lesion	10–12 cores	Transrectal or transperineal	GS ≥ 3 + 4
Klotz 2021 [20]	MRI-fusion TBx	PI-RADS ≥ 3	3.0-T	T2, DWI, DCE	Without	4 per lesion	12 cores	Transrectal	5% or greater chance of GG2 or greater prostate cancer using the Prostate Cancer Prevention Trial Risk Calculator, version 2.
Wei 2023 [9]	MRI-fusion TBx	PI-RADS ≥ 3	1.5-T (Philips)3.0-T (Siemens, Philips)	T2, DWI, DCE, 3D T2	Without	2 per lesion	12 cores	Transrectal	GS of either ≥ 3 + 4 or lesion size > 6 mm.
Panebianco 2015 [13]	MRI-cognitive TBx	PI-RADS ≥ 2	3.0-T (GE)	T2, DWI, DCE	With	2 per lesion	14 cores	Transrectal	GS ≥ 3 + 4
Park 2011 [14]	MRI-cognitive TBx	NR	3.0-T (Philips)	T2, DWI, DCE	Without	NR	10–12 cores	Transrectal	NR
Porpiglia 2023 [10]	MRI-fusion TBx	PI-RADS ≥ 3	1.5-T	T2, DWI, DCE	With	3–6 per lesion	12 cores	Transrectal or transperineal	GS ≥ 7 or MCCL ≥ 5 mm
Porpiglia 2017 [11]	MRI-fusion TBx	PI-RADS > 3	NR	T2, DWI, DCE	NR	4–6 per lesion	12 cores	Transrectal or transperineal	GS ≥ ISUP 2 or maximum CCL ≥ 5 mm
Tonttila 2016 [16]	MRI-cognitive TBx	Likert ≥ 2/4 (1–4 likelihood)	3.0-T (Siemens)	T1, T2, DCE, DWI, ADC	Without	1–2 per lesion	10–12 cores	Transrectal	GS > 3 + 3, >two positive cores, or MCCL ≥ 3 mm
Zhang 2020 [12]	MRI-fusion TBx	PI-RADS ≥ 3	3.0-T	T2, DWI, DCE	NR	2 per lesion	12 cores	Transperineal	at least one core with a Gleason score of 3 + 4, or a score of 6 with a maximum cancer core length 4 mm.

DCE = dynamic contrast enhanced; DWI = diffusion-weighted imaging; ADC = apparent diffusion coefficient; 3D = three-dimensional; mpMRI = multiparametric MRI; MRI = magnetic resonance imaging; NR = not reported; PI-RADS = Prostate Imaging Reporting and Data System; TBx = targeted biopsy; mm = millimetre; T = tesla; PCA = prostate cancer; TRUS-Bx = transrectal ultrasound-guided biopsy; MCCL = maximum cancer core length; GS = Gleason score.

**Table 3:** Subgroup analyses.

Variables	Stratification	Number of Studies	Model	RR (95% CI)	*p* Value	I^2^
MRI machine field strength (Tesla)	1.5 T	2 [10,15]	Random	1.45 [0.53, 3.96]	*p* = 0.46	92%
	3.0 T	5 [12,13,14,16,20]	Random	1.59 [1.16, 2.16]	*p* = 0.004	77%
Endorectal coil	With	2 [10,13]	Fixed	1.99 [1.77, 2.24]	*p* = 0.00001	0%
	Without	5 [9,14,15,16,20]	Random	1.10 [0.92, 1.33]	*p* = 0.29	40%
MRI-TBx cores number	≤2	5 [9,12,13,15,16]	Random	1.33 [0.93, 1.89]	*p* = 0.12	91%
	≥3	4 [10,11,19,20]	Random	1.42 [1.14, 1.77]	*p* = 0.002	61%

MRI = magnetic resonance imaging; T = tesla; TBx = targeted biopsy; RR = risk ratio; CI = confidence interval; *p* = probability value; I^2^ = heterogeneity index.

## Data Availability

This article contains study data.

## Supplementary Materials

The following supporting information can be downloaded at: https://www.mdpi.com/article/10.3390/cancers17030458/s1, Table S1: Population characteristics [9,10,11,12,13,14,15,16,19,20].

## References

[1] Bray F., Ferlay J., Soerjomataram I., Siegel R.L., Torre L.A., Jemal A. (2018). Global cancer statistics 2018: GLOBOCAN estimates of incidence and mortality worldwide for 36 cancers in 185 countries. CA A Cancer J. Clin..

[2] Siegel R.L., Giaquinto A.N., Jemal A. (2024). Cancer statistics, 2024. CA A Cancer J. Clin..

[3] Karakiewicz P.I., Benayoun S., Kattan M.W., Perrotte P., Valiquette L., Scardino P.T., Cagiannos I., Heinzer H., Tanguay S., Aprikian A.G. (2005). Development and validation of a nomogram predicting the outcome of prostate biopsy based on patient age, digital rectal examination and serum prostate specific antigen. J. Urol..

[4] Ahmed H.U., Bosaily A.E.-S., Brown L.C., Gabe R., Kaplan R., Parmar M.K., Collaco-Moraes Y., Ward K., Hindley R.G., Freeman A. (2017). Diagnostic accuracy of multi-parametric MRI and TRUS biopsy in prostate cancer (PROMIS): A paired validating confirmatory study. Lancet.

[5] Harvey C., Pilcher J., Richenberg J., Patel U., Frauscher F. (2012). Applications of transrectal ultrasound in prostate cancer. Br. J. Radiol..

[6] O’Connor L.P., Lebastchi A.H., Horuz R., Rastinehad A.R., Siddiqui M.M., Grummet J., Kastner C., Ahmed H.U., Pinto P.A., Turkbey B. (2021). Role of multiparametric prostate MRI in the management of prostate cancer. World J. Urol..

[7] Bhat K.R.S., Samavedi S., Moschovas M.C., Onol F.F., Roof S., Rogers T., Patel V.R., Sivaraman A. (2021). Magnetic resonance imaging-guided prostate biopsy—A review of literature. Asian J. Urol..

[8] Kasivisvanathan V., Jichi F., Klotz L., Villers A., Taneja S.S., Punwani S., Freeman A., Emberton M., Moore C.M. (2017). A multicentre randomised controlled trial assessing whether MRI-targeted biopsy is non-inferior to standard transrectal ultrasound guided biopsy for the diagnosis of clinically significant prostate cancer in men without prior biopsy: A study protocol. BMJ Open.

[9] Wei C., Szewczyk-Bieda M., Bates A.S., Donnan P.T., Rauchhaus P., Gandy S., Ragupathy S.K.A., Singh P., Coll K., Serhan J. (2023). Multicenter randomized trial assessing MRI and image-guided biopsy for suspected prostate cancer: The MULTIPROS study. Radiology.

[10] Porpiglia F., Checcucci E., Piramide F., Amparore D., Piana A., Volpi G., Granato S., Zamengo D., Stura I., Alladio E. (2023). A prospective randomized controlled trial comparing target prostate biopsy alone approach vs. target plus standard in naïve patients with positive mpMRI. Minerva Urol. Nephrol..

[11] Porpiglia F., Manfredi M., Mele F., Cossu M., Bollito E., Veltri A., Cirillo S., Regge D., Faletti R., Passera R. (2017). Diagnostic pathway with multiparametric magnetic resonance imaging versus standard pathway: Results from a randomized prospective study in biopsy-naïve patients with suspected prostate cancer. Eur. Urol..

[12] Zhang J., Zhu A., Sun D., Guo S., Zhang H., Liu S., Fu Q., Zhang K. (2020). Is targeted magnetic resonance imaging/transrectal ultrasound fusion prostate biopsy enough for the detection of prostate cancer in patients with PI-RADS ≥3: Results of a prospective, randomized clinical trial. J. Cancer. Res. Ther..

[13] Panebianco V., Barchetti F., Sciarra A., Ciardi A., Indino E.L., Papalia R., Gallucci M., Tombolini V., Gentile V., Catalano C. (2015). Multiparametric magnetic resonance imaging vs. standard care in men being evaluated for prostate cancer: A randomized study. Urol. Oncol..

[14] Park B.K., Park J.W., Park S.Y., Kim C.K., Lee H.M., Jeon S.S., Seo S.I., Jeong B.C., Choi H.Y. (2011). Prospective evaluation of 3-T MRI performed before initial transrectal ultrasound-guided prostate biopsy in patients with high prostate-specific antigen and no previous biopsy. AJR Am. J. Roentgenol..

[15] Baco E., Rud E., Eri L.M., Moen G., Vlatkovic L., Svindland A., Eggesbø H.B., Ukimura O. (2016). A Randomized Controlled Trial to Assess and Compare the Outcomes of Two-core Prostate Biopsy Guided by Fused Magnetic Resonance and Transrectal Ultrasound Images and Traditional 12-core Systematic Biopsy. Eur. Urol..

[16] Tonttila P.P., Lantto J., Pääkkö E., Piippo U., Kauppila S., Lammentausta E., Ohtonen P., Vaarala M.H. (2016). Prebiopsy Multiparametric Magnetic Resonance Imaging for Prostate Cancer Diagnosis in Biopsy-naive Men with Suspected Prostate Cancer Based on Elevated Prostate-specific Antigen Values: Results from a Randomized Prospective Blinded Controlled Trial. Eur. Urol..

[17] Xie J., Jin C., Liu M., Sun K., Jin Z., Ding Z., Gong X. (2022). MRI/Transrectal ultrasound fusion-guided targeted biopsy and transrectal ultrasound-guided systematic biopsy for diagnosis of prostate cancer: A systematic review and meta-analysis. Front. Oncol..

[18] Moher D., Liberati A., Tetzlaff J., Altman D.G., PRISMA Group (2009). Preferred reporting items for systematic reviews and meta-analyses: The PRISMA statement. Ann. Intern. Med..

[19] Kasivisvanathan V., Rannikko A.S., Borghi M., Panebianco V., Mynderse L.A., Vaarala M.H., Briganti A., Budäus L., Hellawell G., Hindley R.G. (2018). MRI-Targeted or Standard Biopsy for Prostate-Cancer Diagnosis. N. Engl. J. Med..

[20] Klotz L., Chin J., Black P.C., Finelli A., Anidjar M., Bladou F., Mercado A., Levental M., Ghai S., Chang S.D. (2021). Comparison of multiparametric magnetic resonance imaging–targeted biopsy with systematic transrectal ultrasonography biopsy for biopsy-naive men at risk for prostate cancer: A phase 3 randomized clinical trial. JAMA Oncol..

[21] Hu X., Yang Z.Q., Shao Y.X., Dou W.C., Xiong S.C., Yang W.X., Li X. (2020). MRI-targeted biopsy versus standard transrectal ultrasound-guided biopsy: A systematic review and meta-analysis of randomized controlled trials. Abdom. Radiol..

[22] Woo S., Suh C.H., Eastham J.A., Zelefsky M.J., Morris M.J., Abida W., Scher H.I., Sidlow R., Becker A.S., Wibmer A.G. (2019). Comparison of magnetic resonance imaging-stratified clinical pathways and systematic transrectal ultrasound-guided biopsy pathway for the detection of clinically significant prostate cancer: A systematic review and meta-analysis of randomized controlled trials. Eur. Urol. Oncol..

[23] Pirola G.M., Castellani D., Orecchia L., Giulioni C., Gubbiotti M., Rubilotta E., Maggi M., Teoh J.Y.-C., Gauhar V., Naselli A. (2023). Transperineal US-MRI fusion-guided biopsy for the detection of clinical significant prostate cancer: A systematic review and meta-analysis comparing cognitive and software-assisted technique. Cancers.

[24] Hsieh P.-F., Li P.-I., Lin W.-C., Chang H., Chang C.-H., Wu H.-C., Chang Y.-H., Wang Y.-D., Huang W.-C., Huang C.-P. (2023). Learning curve of transperineal MRI/US fusion prostate biopsy: 4-year experience. Life.

[25] Pokorny M.R., De Rooij M., Duncan E., Schröder F.H., Parkinson R., Barentsz J.O., Thompson L.C. (2014). Prospective study of diagnostic accuracy comparing prostate cancer detection by transrectal ultrasound–guided biopsy versus magnetic resonance (MR) imaging with subsequent MR-guided biopsy in men without previous prostate biopsies. Eur. Urol..

[26] Wegelin O., van Melick H.H., Hooft L., Bosch J.R., Reitsma H.B., Barentsz J.O., Somford D.M. (2017). Comparing three different techniques for magnetic resonance imaging-targeted prostate biopsies: A systematic review of in-bore versus magnetic resonance imaging-transrectal ultrasound fusion versus cognitive registration. Is there a preferred technique?. Eur. Urol..

[27] Bass E.J., Pantovic A., Connor M.J., Loeb S., Rastinehad A.R., Winkler M., Gabe R., Ahmed H.U. (2022). Diagnostic accuracy of magnetic resonance imaging targeted biopsy techniques compared to transrectal ultrasound guided biopsy of the prostate: A systematic review and meta-analysis. Prostate Cancer Prostatic Dis..

[28] El-Helaly H.A.-A., Mahmoud A.A.-A., Magdy A.M., Hasehem A., Ibrahim H.M., Mohamed K.M., Ismail M.H. (2023). Impact of changing PI-RADS cutoff on prostate cancer detection by MRI cognitive fusion biopsy in biopsy-naïve patients. J. Egypt. Natl. Cancer Inst..

[29] Falagario U.G., Jambor I., Lantz A., Ettala O., Stabile A., Taimen P., Aronen H.J., Knaapila J., Perez I.M., Gandaglia G. (2021). Combined use of prostate-specific antigen density and magnetic resonance imaging for prostate biopsy decision planning: A retrospective multi-institutional study using the prostate magnetic resonance imaging outcome database (PROMOD). Eur. Urol. Oncol..

[30] Malshy K., Ochsner A., Homer A., Allu S., Passarelli N., Sojka A., Glebocki R., Golijanin B., Ortiz R., Eaton S. (2024). Consistent predictive ability of prostate-specific antigen density prediction model for clinically significant prostate cancer across age strata. Prostate.

[31] Yusim I., Krenawi M., Mazor E., Novack V., Mabjeesh N.J. (2020). The use of prostate specific antigen density to predict clinically significant prostate cancer. Sci. Rep..

[32] Wang S., Kozarek J., Russell R., Drescher M., Khan A., Kundra V., Barry K.H., Naslund M., Siddiqui M.M. (2024). Diagnostic performance of prostate-specific antigen density for detecting clinically significant prostate cancer in the era of magnetic resonance imaging: A systematic review and meta-analysis. Eur. Urol. Oncol..

