# Supplementary material for: Identifying Optimal Prostate Biopsy Strategy for the Detection Rate of Clinically Significant Prostate Cancer: A Systematic Review and Meta-Analysis of Randomised Controlled Trials (RCTs) in Biopsy-Naïve Population
Source: Cancers (Basel). 2025 Jan 29;17(3):458. doi: 10.3390/cancers17030458 (PMC11816062; doi:10.3390/cancers17030458)
Supplement: Supplementary file 1 [file cancers-17-00458-s001.zip › cancers-3423729-supplementary.pdf]

Supplementary materials:

The search strategy had three main concepts from which the synonyms and different terms for the same concept were added:

1. Concept 1 Prostate biopsy: "Prostate biopsy" OR "prostate cancer" OR "prostatic neoplasms"
2. Concept 2 MRI-guided biopsy: "Image-guid*" OR "MRI guid*" OR "MRI target*" OR "magnetic resonance imaging guid*" OR "magnetic resonance imaging target*" OR MRI-GB OR MRI-TB OR fusion OR "Image guided biopsy" OR "Image-guided biopsy" OR "Targeted biopsy" OR MRI OR "Magnetic resonance imaging"
3. Concept 3 Systematic biopsy: "Ultrasound guid*" OR "ultrasound target*" OR "systematic biopsy" OR SB OR TRUS OR transrectal OR transperineal OR "randomized biopsy" OR "ultrasonography" OR ultrasound
4. Concept 4: RCT OR randomized OR randomise

Table S1- Population characteristics.

Study	Patients ages (years)		PSA level(ng/ml)		Prostate volume (ml)	
	Arm (1)	Arm (2)	Arm (1)	Arm (2)	Arm (1)	Arm (2)
Baco 2016 [15]	Median (IQR) 64 (58–69)	Median (IQR) 65 (59–69)	Median (IQR) 6.9 (5.2–9.2)	Median (IQR) 7.6 (5.9–10.4)	Median (IQR) 45 (33-60)	Median (IQR)40(29-52)
Kasivisvanathan 2018 [19]	Means ±SD 64.4 ± 7.5	Means ±SD 64.5 ± 8.0	Median (IQR) 6.75 (5.16– 9.35)	Median (IQR) 6.50 (5.14–8.65)	NR	NR
Klotz 2021 [20]	Mean (SD) 65.3(7.6)	Mean (SD) 64.5(8.8)	Mean (SD) 7.5(3.6)	Mean (SD) 6.3(3.0)	Mean (SD) 60(45)	Mean (SD) 48(25)
Wei 2023 [9]	mean (SD) 65.4 ± 6.3	mean (SD) 65.1± 6.2	mean (SD) 9.3 ± 6.1	mean (SD) 10.2 ± 8.6	NR	NR
Panebianco 2015 [13]	Median (IQR) 63 (51–80)	Median (IQR) 64 (53–82)	Median (IQR) 5.8(4.8-18)	Median (IQR) 5.2(4.5-16.5)	NR	NR
Park 2011 [14]	Mean 63 (40–82)	Mean 61 (37–92)	Mean 6.1 (4.0–9.7)	Mean 5.6 (2.9–9.9)	Mean 37(17-94)	Mean 38(15-87)
Porpiglia 2023 [10]	Median (IQR) 64 (58–70)	Median (IQR) 66 (60–70)	Median (IQR) 5.9 (4.8–7.5)	Median (IQR) 6.7 (5.5–8.5)	Median (IQR) 46.2 (34.5-71.6)	Median (IQR) 45.7 (34.6-65.0)
Porpiglia 2017 [11]	mean (95%CI) 68,5 (63,0-74,0)	mean (95%CI) 68,1 (63,0-74,0)	mean (95%CI) PSA, ng/dL 7,2 (4,9-8,8)	mean (95%CI) PSA, ng/dL 7,0 (4,9-8,5)	mean (95%CI) CC. 51,3 (32,1-60,5)	mean (95%CI) CC. 50,2 (32,9-61,5)

Tonttila 2016 [16]	Median (IQR) 63 (60–66)	Median (IQR)62 (56–67)	Median (IQR)6.1 (4.2–9.9)	Median (IQR) 6.2 (4.0–10.7)	Median (IQR) 27.8 (23.5-36.6)	Median (IQR) 31.8 (26.1-44.3)
Zhang 2020 [12]	65.15±5.80(49-75)	64.44±5.40 (46-74)	18.73±11.87 (5.22-46.58)	20.73±11.56 (5.16-48.81)	47.65±20.33 (20.44-98.57)	51.01±19.59 (22.38-96.33)

IQR = interquartile range; SD = standard deviation; PSA = prostate-specific antigen; ng = nanogram; ml = millilitre; NR = not reported; CI = confidence interval; dL= decilitre
